# Design and Characterisation of Titanium Nitride Subarrays of Kinetic Inductance Detectors for Passive Terahertz Imaging

**DOI:** 10.1007/s10909-018-2023-z

**Published:** 2018-07-18

**Authors:** Dmitry Morozov, Simon M. Doyle, Archan Banerjee, Thomas L. R. Brien, Dilini Hemakumara, Iain G. Thayne, Ken Wood, Robert H. Hadfield

**Affiliations:** 1 School of Engineering, University of Glasgow, Glasgow, UK; 2 School of Physics and Astronomy, Cardiff University, Cardiff, UK; 3 QMC Instruments, Cardiff, UK

**Keywords:** Kinetic inductance detector, Titanium nitride, ALD

## Abstract

We report on the investigation of titanium nitride (TiN) thin films deposited via atomic layer deposition (ALD) for microwave kinetic inductance detectors (MKID). Using our in-house ALD process, we have grown a sequence of TiN thin films (thickness 15, 30, 60 nm). The films have been characterised in terms of superconducting transition temperature $T_\mathrm{c}$, sheet resistance $R_\mathrm{s}$ and microstructure. We have fabricated test resonator structures and characterised them at a temperature of 300 mK. At 350 GHz, we report an optical noise equivalent power $\hbox {NEP}_\mathrm{opt} \approx 2.3\times 10^{-15}~\hbox {W}/\sqrt{\hbox {Hz}}$, which is promising for passive terahertz imaging applications.

## Introduction

THz imaging for security applications attracts increasing interest in both the research community and industrial sector. Several passive imagers have been built utilising different types of superconducting bolometers and semiconducting detectors [1, 2]. However, developments in THz astronomy instrumentation have resulted in the realisation of state-of-the-art arrays of sensitive detectors for space- and ground-based astronomical observatories [3–5]. MKIDs have the advantage of relatively simple fabrication and scalability which makes them a technology of choice for building an imager with large numbers of pixels [6]. Recently, arrays of MKIDs, predominantly used in astronomical instruments, were successfully used in a THz camera built by Cardiff University [7]. With lumped-element MKIDs (LEKIDs) based on Al films, a demonstration camera achieved quasi-video frame rate of 2 Hz and noise equivalent differential temperature, $\hbox {NEDT} \approx 0.1$ K at 350 GHz. At the same time, usage of Al-based chips requires sub-300 mK cryogenic set-up, which adds complexity to the overall design. On the other hand, TiN film-based MKIDs with resistivity $>100~\upmu \Omega \mathrm{cm}$ and $T_\mathrm{c} = 2{-}4~\hbox {K}$ can be operated at $>0.3~\hbox {K}$ bath temperatures, which is easily achievable with modern compact cryogenic systems. For practical applications in security, image rate of 25 frames per second with NEDT $\le$ 0.1 K per frame is desirable. For a 350 GHz imager with linear array of detectors, similar to the one reported in [7], each pixel needs to have a time constant of $\le$ 100 ${ \upmu \mathrm{s}}$ and photon-noise-limited NEP with the optical load of few hundreds pW, where exact values depend on the system’s optical bandwidth and background temperature. TiN resonator devices were characterised previously by a number of groups. Reported results were obtained both with sputtered TiN films and with films made by an ALD process [8, 12]. Study of electrodynamic response shows that the behaviour of TiN films deviates from Mattis–Bardeen theory [10] and temperature dependence of microwave-probed relaxation time has weaker temperature dependence than predicted in Kaplan theory [9]. At the same time, relaxation times measured in both sputtered and ALD TiN films are close to show similar values and temperature dependencies [13, 14]. Although TiN exhibits anomalous behaviour, it remains promising for applications in high background THz detection. TiN films have high $R_\mathrm{s}$, which is favourable for direct absorption of EM radiation in compact designs. High kinetic inductance per square ($L_\mathrm{k}$) of TiN allows operating with lower frequency read-out electronics, which is more preferable in commercial systems. Adjustability of $T_\mathrm{c}$ and $R_\mathrm{s}$ through growth parameters is another advantage of TiN. ALD is a recent technique which uses a self-limiting reactions of gas precursors at the film surface [15–18]. ALD films for previous microwave resonator studies were made with inorganic $\hbox {TiCl}_4$ precursor and a mix of $\hbox {H}_2/\hbox {N}_2$ plasma reactive gases. Drawbacks of inorganic-based ALD are high deposition temperature and chlorine contamination. Metalorganic precursors, such as TDMAT (tetrakis dimethylamino titanium, $\hbox {Ti}(\hbox {N}(\hbox {CH}_3)_2)_4$), were studied as an alternative to $\hbox {TiCl}_4$. On the one hand, usage of TDMAT and $\hbox {NH}_3$ precursors allowed lower growth temperatures, and on the other hand, high resistivity and carbon contamination were initial concerns. Plasma enhancement and usage of TDMAT and $\hbox {N}_2/\hbox {H}_2$ precursors greatly improve TiN quality [15]. Moreover, the self-limiting nature of ALD ensures greater control over film thickness and better uniformity over the film surface. Here, we report on early results of a development of TiN MKID array for terrestrial 350 GHz camera. We present: (a) results of deposition and characterisation of ALD films which were used to design and fabricate MKID subarrays, (b) results on optical performance at 300 mK.

**Fig. 1:**
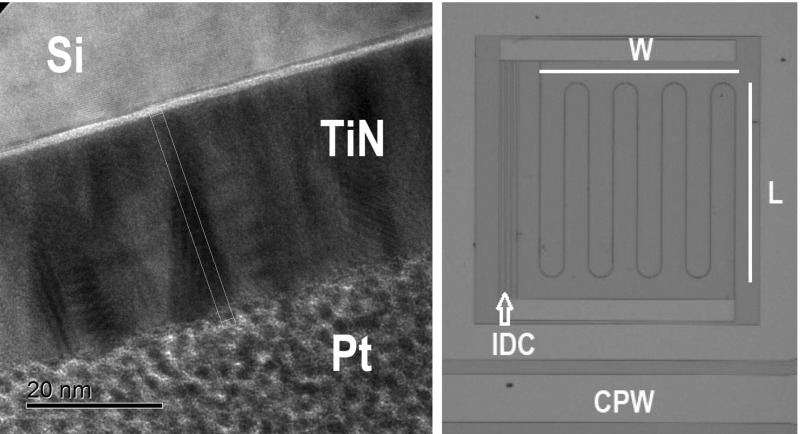
(Left): TEM cross-sectional image of 30-nm ALD film, (Right): Optical micrograph of the fabricated MKID device (Colour figure online)

## Film fabrication, subarray design and experimental techniques

TiN films for this study were grown in the ALD system at University of Glasgow (Oxford Instruments FlexAL). We used high-resistivity ($>10^4~{\Omega \mathrm{cm}}$) Si (111) substrates dipped into buffered HF (with $\hbox {NH}_4\hbox {F}$ as 1:10) diluted with water as 1:20 for 2 min 30 s. Films were grown with TDMAT and $\hbox {H}_2/\hbox {N}_2$ plasma. Before the deposition process, Si substrates were heated up to 350 $^\circ$C. The ALD process consists of a number of repeated cycles with each cycle resulting in deposition of one layer of material. Cycles consist of exposure to precursors, purging, exposure to reactive gas and plasma and purging. The film thickness was defined by the number of cycles. To deposit a 30-nm-thick film, 375 cycles were needed. Growth lasted 240 min, with rate of 0.125 nm/min or 0.08 nm/cycle. We have deposited a set of films 15, 30 and 60 nm thick. Cross sections of the films were imaged with transmission electron microscope (TEM) as seen in Fig. 1 (Left). Columnar structure of TiN is seen as dark areas. Film thickness (*d*), resistivity (${\rho }$), $\hbox {R}_\mathrm{s}$ and $T_\mathrm{c}$ of the films are shown in Table 1. We have designed a test subarray consisting of 12 MKID pixels with varying geometries and coupling factors. In this paper, we report the results obtained with large pixel fabricated with ALD4 film. Devices consist of an inductive meander in series with an interdigital capacitor (IDC) capacitively coupled to a $50~{\Omega }$ coplanar waveguide (CPW). The meander of length (*L*) and width (*W*) is made up of a line $3~{\upmu \mathrm{m}}$ wide. For the optically tested device, a distance between the lines is equal to 120 ${\upmu \mathrm{m}}$ and covers the area of 987$\times 987~{\upmu \mathrm{m}}^2$. Optical photograph of the device is shown in Fig. 1 (Right). Kinetic inductance per square, $L{_\mathrm{k}} = 22.2$ pH/sq, was derived from comparison of measured value of $f_\mathrm{res}$ and simulated $f_\mathrm{res}$ with $L{_\mathrm{k}} = 0$, noting that full inductance of the meander is $L=L_\mathrm{g}+L_\mathrm{k}$, where $L_\mathrm{g}$ is geometric inductance. From Mattis–Bardeen theory, $L_\mathrm{k}$ can be predicted as $L_\mathrm{k}\approx (R_\mathrm{s}\hbar )/(\pi \varDelta ) \approx 29$ pH/sq for ALD4 film with $R_{\mathrm{s}} = 43.5~{\Omega / \mathrm{sq}}$; here, $\hbar$ is the reduced Planck constant and $\varDelta$ is the energy gap. Absorption of EM radiation in this non-optimised MKID device was simulated using HFSS software. At 350 GHz, simulated optical efficiency is equal to $\eta _\mathrm{opt} \approx 8\%$. The absorption was normalised to the power received by meander section in both polarisations. Devices were patterned by direct photolithography and dry etch with $\hbox {Cl}_2$/Ar plasma. The chip was installed into the $^3$He cryostat with base temperature of 230–900 mK. Transmission of the microwave signal through the chip is amplified by a cold low noise amplifier with bandwidth of 10 GHz and noise figure of $\approx$ 6 K. Forward transmission was measured with both vector network analyser (VNA) and homodyne read-out as $S_{21} = I + \mathrm{i}Q$. For noise measurements with a fixed tone, the output signal was sampled with the rate of 200 kHz. In order to extract resonance parameters, we fit transmission data as $S_{21} = 1 - \frac{Q_\mathrm{r}}{Q_\mathrm{c}}\left( 1 + 2\mathrm{i}Q_\mathrm{r} \left( \frac{f - f_\mathrm{res}}{f_\mathrm{res}}\right) \right) ^{-1}$, where $Q_\mathrm{c}$ and $Q_\mathrm{r}$ are the coupling and overall quality factors of the resonator, respectively. The array was illuminated with a blackbody source thermally suspended from a 4 K shield. The filter stack in front of the blackbody consists of thermal blocks and band defining metal mesh filters. Transmission band is centred at 350 GHz with $\approx$ 10% bandwidth. Optical power ($P_\mathrm{opt}$) incident on the MKID device was calculated in filter band taking into account a solid angle. In our tests, the blackbody temperature ($T_\mathrm{bb}$) was in the range of 5.3–250 K, corresponding to $P_\mathrm{opt}\approx 1 - 297$ pW.

**Table 1:** DC parameters of films

Film	*d* (nm)	$\rho ~({\upmu \Omega \mathrm{cm}})$	$R_\mathrm{s}~({\Omega /\mathrm{sq}})$	$T_\mathrm{c}$ (K)
ALD1	30	449	149	2.4
ALD2	30	432	139	2.09
ALD3	15	270	180	2.04
ALD4	60	195	43.5	2.06

## Measurements

**Fig. 2:**
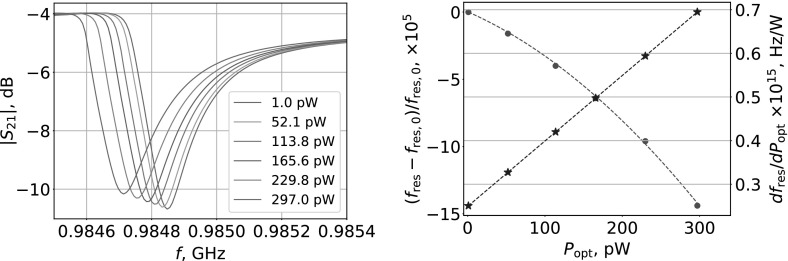
(Left): $S_\mathrm{21}$ transmission at different $P_\mathrm{opt}$, (Right): response of the $f_\mathrm{res}$ to the blackbody power (green circles, left) and responsivity (blue stars, right) (Colour figure online)

**Fig. 3:**
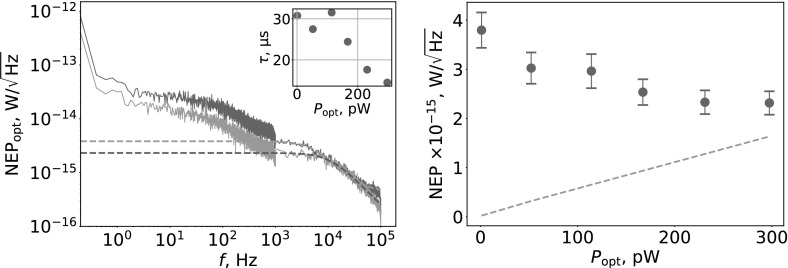
(Left): $\mathrm{NEP_{opt}}$ for $P_\mathrm{opt}\approx$ 1 pW (blue) and 297 pW (green), dashed lines are fit to the data. (Inset Left): dependence of time constant on $P_\mathrm{opt}$. (Right): $\mathrm{NEP_{opt}}$ versus $P_\mathrm{opt}$ (blue circles), $\mathrm{NEP_{photon}}$ versus $P_\mathrm{opt}$ (dashed orange line) (Colour figure online)

Measurements were performed at 300 mK with $T_\mathrm{b}/T_\mathrm{c}\approx 0.14$. Firstly, we performed frequency sweeps of $S_\mathrm{21}$ with VNA to find values of $f_\mathrm{res}$ and resonator internal quality factor, $Q_\mathrm{i}$. ALD4 device has $Q_\mathrm{i}\approx 2.3\times 10^4$, $Q_\mathrm{r}\approx 4.6\times 10^3$ and $Q_\mathrm{c}\approx 5.8\times 10^3$ at the lowest $T_\mathrm{bb}$. Value of $Q_\mathrm{i}$ reduces to $\approx 1.9\times 10^4$ at $T_\mathrm{bb} = 250$ K. Secondly, we performed a frequency sweep of *I* and *Q* near $f_\mathrm{res}$ with homodyne read-out to extract parameters needed for calculation of frequency perturbations $\delta f(t)$. Finally, the tone was fixed at $f_\mathrm{res}$ and time streams of *I* and *Q* data were recorded and converted into $\delta f(t)$ according to [19]: $\delta f(t) = \left( I(t)\frac{\mathrm{d}I}{\mathrm{d}f}+Q(t)\frac{\mathrm{d}Q}{\mathrm{d}f}\right) \times \left( \left( \frac{\mathrm{d}I}{\mathrm{d}f}\right) ^2+\left( \frac{\mathrm{d}Q}{\mathrm{d}f}\right) ^2\right) ^{-1}$. Absorption of in-band radiation results in the shift of $f_\mathrm{res}$ and decrease of $Q_\mathrm{i}$ relative to equilibrium state. $S_\mathrm{21}$ for MKID device exposed to different $P_\mathrm{opt}$ is shown in Fig. 2 (Left). A fractional change $\frac{f_\mathrm{res}-f_\mathrm{res,0}}{f_\mathrm{res,0}}$ with increasing $P_\mathrm{opt}$, where $f_\mathrm{res,0}$ is resonance frequency at lowest $T_\mathrm{bb}$, is shown in Fig. 2 (Right). Optical responsivity of the device was found as $\mathrm{d}f_\mathrm{res}/\mathrm{d}P_\mathrm{opt}$. It increases with increasing $P_\mathrm{opt}$ as seen in Fig. 2 (Right). Similar behaviour of amplitude responsivity was observed by authors of Ref. [11]. This is opposite to the behaviour of responsivity observed in Al MKIDs [20] where reduction of quasiparticle lifetime at higher quasiparticle densities causes responsivity to decrease with increasing $P_\mathrm{opt}$. As noted by authors of Ref. [11], such anomalous behaviour is possibly due to spatial non-uniformity of superconducting gap energy in highly disordered TiN films. According to this conjecture, at lower optical powers, excess quasiparticles are trapped in low-gap areas, but with increasing optical powers, their freedom to move increases. Optical noise equivalent power can be found as $\mathrm{NEP_{opt}}(f) = \sqrt{S_f(f)}\left( \frac{\mathrm{d}f_\mathrm{res}}{\mathrm{d}P_\mathrm{opt}}\right) ^{-1}$, where $S_f(f)$ is power spectral density of $\delta f(t)$. We have measured $S_f$ as a function of $P_\mathrm{opt}$ at the base temperature of 300 mK and converted it to $\mathrm{NEP_{opt}}$ [Fig. 3 (Left)]. Noise data show the presence of frequency-dependent components at lower frequency part of the spectra which we attribute to read-out system noise. Although raw $S_f(f)$ levels are rising with increasing $P_\mathrm{opt}$, values of $\mathrm{NEP_{opt}}$ are decreasing due to increasing response. Device time constant $\tau$ was found from fits to $\mathrm{NEP_{opt}}$, as shown in Fig. 3 (Left inset). Time $\tau$ is falling from  31 to 14.5 ${\upmu \mathrm{s}}$ for increasing $P_\mathrm{opt}$ from 1 to 297 pW. For comparison, a resonator ring time is $\tau _\mathrm{res} = Q_\mathrm{r}/({\pi }f_\mathrm{res}) \approx 1.5~{\upmu \mathrm{s}}$ with $Q_\mathrm{r}\approx 4.6 \times 10^{3}$. Ideally, intrinsic noise of the detector should be below the level of source noise [21]: $\mathrm{NEP_{photon}} = \sqrt{2P_\mathrm{opt}\mathrm{h}\nu + \frac{2P_\mathrm{opt}^2}{\mathrm{m}{\varDelta }\nu }}$, where $\mathrm{h}$ is the Planck constant, $\nu$ is the central frequency of the source, ${\varDelta } \nu$ is the bandwidth and $m=2$. The terms are a photon shot noise and a wave noise. In Fig. 3 (Right), we plot $\mathrm{NEP_{photon}}$ to compare with $\mathrm{NEP_{opt}}$ obtained at 1 kHz. Measured values of $\mathrm{NEP_{opt}}$ are equal to $\approx 2.3\times 10^{-15}~\mathrm{W/\sqrt{Hz}}$ at $P_\mathrm{opt} > 229$ pW. Photon-noise-limited performance was previously reported for TiN/Ti/TiN MKIDs  [22]. Authors of Ref. [11] reported $\mathrm{NEP_{opt}}$ of TiN MKID, which was roughly an order of magnitude higher than predicted $\mathrm{NEP_{photon}}$. Although our measurements were made with much higher $P_\mathrm{opt}$ levels than in Refs. [11, 22], we can relate our results by comparing received power density, $P_\mathrm{opt}/V$, where *V* is the volume of MKID. In Ref. [22], $P_\mathrm{opt}/V$ is ranging from 0.01 to 0.24 $\mathrm{pW/\upmu m^3}$ for $P_\mathrm{opt}$ in the range of 1 to 21 pW. In Ref. [11], $P_\mathrm{opt}/V = 5.7\times 10^{-4}~\mathrm{pW/\upmu m^3}$ at $P_\mathrm{opt} = 9$ pW. In our experiments, $\mathrm{NEP_{opt}}$ is closest to $\mathrm{NEP_{photon}} = 1.6\times 10^{-15}~\mathrm{W/\sqrt{Hz}}$ at $P_\mathrm{opt} = 297$ pW, where we find $P_\mathrm{opt}/V = 0.16~\mathrm{pW/\upmu m^3}$, which is between the values from Refs. [11, 22].

## Conclusion

We have demonstrated prototype TiN MKID subarray, which at 297 pW of optical load power has $\mathrm{NEP_{opt}}\approx 2.3\times 10^{-15}~\mathrm{W/\sqrt{Hz}}$ and $\tau \approx 31~{\upmu \mathrm{s}}$. While the measured $\tau$ satisfies the requirement of a scanning passive THz imaging system ($<100~{\upmu \mathrm{s}}$), the detector NEP is still higher than the photon noise of the source ($1.6\times 10^{-15}~\mathrm{W/\sqrt{Hz}}$). It is worth noting that the optical load and the value of $\mathrm{NEP_{photon}}$ were defined by the blackbody source temperature of 250 K and by properties of the optical filter. Optimisation of pixel design, in particular inductor geometry and fill factor, for better detector efficiency and improvements of read-out noise at frequency below 1 kHz are required to achieve a source-noise-limited performance. This initial investigation indicates that ALD TiN films are a promising starting point for MKID arrays for applications such as passive terahertz imaging. In contrast to the most commonly used MKID material, Al, TiN can be tuned to achieve higher $T_\mathrm{c}$. We believe that with further optimisation through the ALD growth process, TiN MKID arrays for 1 K operation should be within reach.
